# The transition from medical to vocational rehabilitation in Germany: an exploratory mixed methods study on rehabilitants’ experiences

**DOI:** 10.3389/fresc.2026.1658258

**Published:** 2026-08-05

**Authors:** Eileen Wengemuth, Lukas Kühn, Lara Kleist, Kyung-Eun Anna Choi

**Affiliations:** 1 Center for Health Services Research, Brandenburg Medical School Theodor Fontane, Berlin, Germany; 2 Alice-Salomon-Hochschule Berlin, Berlin, Germany; 3 Department of Therapeutic Health Professions, University Hospital Münster, Münster, Germany; 4 Health Services Research Group, Research Center Medical Imaging and Artificial Intelligence (MIAAI), Danube Private University (DPU) GmbH, Krems-Stein, Austria; 5 Evidence-Based Practice in Brandenburg—A JBI Affiliated Group, Brandenburg, Germany

**Keywords:** autonomy, care transitions, continuity of care, medical rehabilitation, multiprofessional collaboration, occupational rehabilitation, vocational rehabilitation

## Abstract

**Introduction:**

Medical and vocational rehabilitation are both part of the German rehabilitation system. Both aim to improve the functioning and social participation of people with chronic conditions. Vocational rehabilitation, especially, is intended to enable individuals to participate in working life. These two types of rehabilitation settings are not systematically linked, and, as is the case with other transitions of care, this often poses a challenge for rehabilitants. This study aimed to explore the experiences of individuals transitioning from medical to vocational rehabilitation in Germany. We wanted to investigate how and from whom they received information and support, and how these were tailored to meet their needs of autonomy and self-determination.

**Methods:**

We employed a convergent parallel mixed methods design, conducting a survey comprising standardized and self-developed items, as well as semi-structured guided interviews with the same topics as the questionnaire, to gather a more in-depth account of the experiences of rehabilitants. For the quantitative data, we performed descriptive analysis and calculated response frequencies and means. We analyzed the interview data using qualitative content analysis with deductive-inductive coding. The qualitative data were used to provide a more in-depth and descriptive understanding of the survey findings and to ensure that we would not exclude potentially new and relevant topics.

**Results:**

Sixty-one vocational rehabilitants (29f, 30 m, 1d) who had previously completed a medical rehabilitation completed the survey, and seven (6f, 1 m) participated in an interview. The quantitative results indicate that approximately 50% of the rehabilitants reported having been informed about vocational rehabilitation during their medical rehabilitation. Still, fewer reported having received practical support, such as assistance with the application process. General practicioners and specialized physicians were perceived as supportive by approximately 60%; previous or current employers were perceived as least supportive. Regarding autonomy, almost two-thirds of the participants felt self-determined in choosing and starting their vocational rehabilitation program. However, a few reported feeling pressured. Regarding the qualitative data, deductively developed themes included the rehabilitants’ experiences of autonomy, of perceived and desired professional support, and of received information. An inductively developed recurring topic was the experience with the rehabilitation system, which required high navigation skills.

**Discussion:**

Both support and autonomy are vital for rehabilitants at the transition from medical to vocational rehabilitation. According to the participants, the federal employment agency and employers do not currently provide sufficient support. Generally, the lack of cooperation between different stakeholders and institutions complicates the transition. While many rehabilitants reported a satisfying degree of autonomy, there is still room for improvement to put the ideal of self-determination into practice.

## 1 Introduction

Transitions of care between different healthcare settings pose challenges and potential risks, such as worsening of symptoms or re-hospitalization for patients and their families (1–3). Those transitions include patient referrals from a hospital to a long-term care facility, as well as the initiation of outpatient care. The (supported) reintegration into the labor market, such as the old workplace or a retraining program, can also be considered a transition. This points to the importance of ensuring as much continuity of care as possible in integrated, multisectoral healthcare or rehabilitation systems. Continuity of care originally refers to a continuous relationship with one healthcare provider, but it can also be achieved through multidisciplinary teamwork among different health professionals (4). When patients experience continuity of care, this is related to better clinical outcomes, lower costs, and lower risks for hospitalization and death in patients with chronic conditions (5, 6).

In Germany, the rehabilitation system is organized much more independently from other healthcare sectors than in other countries, due to different funders and historical origins. While both are provided for persons with long-lasting health restrictions endangering their ability to work, medical and vocational rehabilitation, although very often delivered consecutively, are unconnected services provided by different institutions (7, 8). Medical rehabilitation is typically delivered in an inpatient setting at rehabilitation clinics specialized for specific diagnosis groups, lasts between 3 and 6 weeks, and is primarily funded by pension funds. Vocational rehabilitation, on the other hand, often has an outpatient setting and is financed mostly by the federal employment agency or pension funds. In Germany, vocational rehabilitation is an umbrella term for a wide range of different services from short diagnostic programs assessing a person's health restrictions and abilities, supported employment, to two-year retraining programs during which rehabilitants learn a new profession (9). Rehabilitants must apply for both medical and vocational rehabilitation by themselves. The rehabilitants' need for vocational rehabilitation is routinely assessed during medical rehabilitation, and vocational rehabilitation often follows medical rehabilitation. It can thus be considered beneficial for rehabilitants to receive professional support in choosing and applying for vocational rehabilitation already during their time in medical rehabilitation. This helps to ensure a smoother transition, allowing rehabilitants who might benefit from it to start the new program without significant delays and to gain more from their vocational rehabilitation (9, 10). However, it is unclear to which extent this is actually happening in practice.

Vocational rehabilitation, such as retraining programs, can aid in returning to and remaining at work, and may even lead to increased subsequent salary (11). Applying for vocational programs is often perceived as complicated by those affected; information gaps contribute to the rehabilitants' uncertainty of whether they are eligible for the programs. Evidence shows that support by health professionals is generally an essential facilitator for rehabilitants' intention to apply for and use of medical rehabilitation (12, 13). It can thus be assumed that continuous professional support during the transition from medical to vocational rehabilitation is also a crucial factor in the use of vocational rehabilitation by rehabilitants. This would include providing information about different possible programs and assisting rehabilitants in choosing and applying for one.

To provide support for a successful rehabilitation, it is crucial to clarify rehabilitants' expectations. This helps them gain a realistic understanding of what expectations can be fulfilled. Additionally, it allows for the possibility of tailoring interventions to align with their expectations, thereby increasing their motivation to adhere to the rehabilitation process (14). Whether or not rehabilitants at the transition between medical and vocational rehabilitation desire and receive the support and information fitting their needs and expectations is a question to be empirically investigated.

The legal basis for rehabilitation in Germany has recently been updated in the form of the Federal Participation Act (15), which aims to enhance the participation and self-determination of persons with disabilities. This also emphasizes the importance of patient autonomy, which means a person decides and acts according to their own preferences (16). Aside from patient autonomy being of ethical value and an inbuilt goal of rehabilitation (17), shared decision-making also leads to lower healthcare costs, and the accommodation of patient preferences is related to better clinical outcomes (18, 19). The German Social Code grants rehabilitants autonomy in the form of meeting their “legitimate wishes” in choosing their rehabilitation programs and settings (20).

A review shows that different aspects of medical in-patient rehabilitation, such as the rehabilitation system, the staff and other patients have the potential to endanger and/ or preserve patient autonomy (17). Also, studies show the importance of professional support for the use of medical rehabilitation (12, 13). However, there is, as of yet, no study that investigates support and autonomy at the transition from medical to vocational rehabilitation. This is also needed to identify potential gaps which could be addressed by the different stakeholders in order to provide rehabilitants with as continuous care as possible.

This study aimed to explore the experiences of rehabilitants during the transition from medical to vocational rehabilitation, as well as to investigate the sources, providers, and means through which rehabilitants received information and support. We were specifically interested in how information and support that was perceived as beneficial were designed to meet the needs of rehabilitants and thus increase their autonomy. Specifically, we asked: What are the experiences and expectations of rehabilitants during the transition between medical and vocational rehabilitation?

## 2 Material and methods

### 2.1 Research design

This study is part of the REHA-Routes research project (resource-oriented support for participation in working life, funded by the German Pension Fund Berlin-Brandenburg 2021-2025, 10-R-40.07.05.07.022), which examines the transition between medical and vocational rehabilitation. Other work packages include exploring the perspectives of service providers [see (21)] and analyzing routine data from the German Pension Fund. To explore the experiences and expectations of rehabilitants, we adopted a convergent parallel mixed methods design as outlined by Creswell and Plano Clark (22).

To capture the rehabilitants' perspective both qualitatively and quantitatively, we first developed an interview guide and a questionnaire. Both were developed at the same time and reviewed by the entire research team to try and cover the research questions and interest as much as possible. The interview guide included thematic sections focusing on the participants' current health and work situations, their expectations regarding current, past, and future rehabilitation services, as well as their experiences with these services. Special emphasis was placed on their experiences related to receiving information, support, and their sense of autonomy and self-determination. Also, we kept some broader narration-inducing questions to enable the participants to bring their own topics into the interviews.

The quantitative questionnaire consisted of 89 items (for medical rehabilitants) or 116 items (for vocational rehabilitants), with a mostly Likert-scale response format. We used mostly self-developed non-validated items to assess the same themes as the interviews (see above). Additionally, the questionnaire included German versions of validated measures, the Work Ability Index [WAI (23)], the 16-item version of the Health Literacy Questionnaire [HLS-EU-Q16 (24)], and the Work & Social Adjustment Scale [WSAS (25)] as we hypothesized that subjectively assessed current and future work ability, health literacy and impaired functioning could be related to how rehabilitants experience the transition between medical and vocational rehabilitation.

The interview guide and questionnaire were designed with slight differences for participants in medical and vocational rehabilitation. For medical rehabilitants, the questions included their intentions to apply for vocational rehabilitation and their expectations. In contrast, for vocational rehabilitants, the focus was on their experiences with the transition between medical and vocational rehabilitation. Both the original interview guide and the questionnaire can be found in the supplemental material.

### 2.2 Sampling and recruitment

For both the interview and survey study, we employed a convenience sampling strategy, approaching rehabilitants from various diagnostic groups aged 18 to 60 who were currently undergoing either medical or vocational rehabilitation at collaborating institutions in the German region Berlin-Brandenburg. The institutions included three medical rehabilitation clinics with different specializations offering inpatient rehabilitation and two vocational rehabilitation centers offering a range of different outpatient vocational interventions funded both by pension funds and the federal employment agency. We displayed posters and distributed leaflets in all institutions. Additionally, in two of these institutions, we organized information events where we provided a brief introduction to the study and invited rehabilitants to participate. Participants who had completed the questionnaire were afterwards shown another invitation to contact us if they were interested in participating in an interview.

In line with the study's objectives, the eligibility criteria for rehabilitants in medical rehabilitation included experiencing work-related issues and having an interest in attending a vocational rehabilitation program afterward. Vocational rehabilitants were eligible if they had completed medical rehabilitation prior to their current vocational rehabilitation. All participants provided their written informed consent. The consent forms differed slightly for interview and survey participants.

### 2.3 Data collection

Quantitative: The surveys were completed between May and December 2023. The long data collection period was due to the low response rate and then subsequently planned in-person visits. Participants could either answer the survey straight away or later and then submit it electronically or by post. They had the option to choose between a paper-pencil format and an online version using LimeSurvey. The paper-pencil responses were later digitized. All survey data was collected anonymously.

Qualitative: EW, a female psychologist, conducted all interviews one-on-one via telephone in October and November 2023. Some of the interviewees had seen her before when she was visiting the rehabilitation center to recruit participants. Apart from that and a brief introduction at the beginning of each interview, there was no prior relationship. The interviews were audio recorded, transcribed, and anonymized. They took between 35 and 50 min. All participants were interviewed once. All interviews were conducted in German.

### 2.4 Data analysis

We analyzed the interviews using qualitative content analysis (26). Codes were developed both deductively, based on the study's research questions and the interview guide, and inductively, to ensure that the perspectives of the rehabilitants were adequately represented. EW, the first author, created the coding frame and then discussed it with the other authors of this article who have different disciplinary backgrounds, including health services research (LaK, LK, AC), rehabilitation studies (LaK), physiotherapy (LK), and psychology (AC). The coding frame was revised accordingly. To ensure inter-coder congruity, excerpts from the interviews were also coded by and discussed with the members of a qualitative methods workshop set at the Center for Health Services Research Brandenburg. When reporting the interview results, we followed the COREQ (Consolidated criteria for reporting qualitative research) checklist for reporting qualitative data, which can be found in the appendix (27).

We used the software MAXQDA for organizing the codes and interview transcripts.

For the survey data, we conducted descriptive analysis in SPSS, calculating means and response frequencies. For each item, we analyzed the existing data. Statistical approximation methods accounting for missing values were not used. Due to the relatively small sample size and lacking data from medical rehabilitation, we did not perform further analyses or group comparisons and did not use the data from the validated measures.

Afterwards, we brought together the quantitative and qualitative results from the different thematic blocks to check for the distribution and potential generalizability of the qualitatively generated results as well as a more in-depth meanings and contexts of the quantitatively generated results.

## 3 Results

### 3.1 Participant characteristics

Quantitative: Sixty-one vocational rehabilitants who were at the time of the participation enrolled in a vocational rehabilitation intervention and thirteen medical rehabilitants participated in the survey. Due to the small response rate in medical rehabilitation clinics, medical rehabilitants were excluded from this analysis (see discussion below). Table 1 provides an overview of the sociodemographic and illness-related data for the included survey participants. The time between applying for and starting a vocational rehabilitation program ranged from less than one month to several years among the included survey participants, with a mean of 11 months.

**Table 1:** Sociodemographic and illness-related data of survey participants.

Characteristic	Options	Values/ numbers (*N* = 61)
Age		Mean 43,3 years; SD 7,9
Gender	female	29
	male	31
	diverse	1
Net income per month	Less than 1.000 €	8
	1.000–1.500 €	26
	1.500–2.500 €	19
	More than 2.500 €	6
	No reply:	2
Education (general schooling)	Secondary school degree (9 years)	8
	Intermediate school degree (10 years)	39
	Grammar school degree (13 years)	14
Professional education	Vocational qualification	43
	Skilled worker qualification	4
	University degree	6
	Other	1
	No professional degree	7
Main illness groups/ affected systems (more than one could be selected)	Cardiovascular	13
	Mental health	37
	Musculoskeletal	26
	Addiction	8
	Cancer	4
	Digestive/ Gastroenterological	6
	Neural/ Brain	4
	Skin	4
	Metabolism	6
	Respiratory	8

Qualitative: Seven rehabilitants who were attending a vocational rehabilitation program at the time volunteered and participated in the interviews. Six of them were female and one was male. They were aged between 29 and 57 and had been working for 11–40 years. Four of them an intermediate (10 years) school degree, one had had nine years of schooling and two had a grammar school degree. They had been treated for mental health issues (4), musculoskeletal (4), and gastroenterological (2) illnesses, several of them for more than one diagnosis group. Five of them were at the time of the interview participating in a full retraining intervention which will provide them with a new qualification. Two of them were in qualification interventions with slightly lower requirements specifically designed for people with mental health issues. No other rehabilitants from vocational rehabilitation and none from medical rehabilitation volunteered to take part in an interview.

### 3.2 Information on & support for applying for vocational rehabilitation during medical rehabilitation

**Figure 1:**
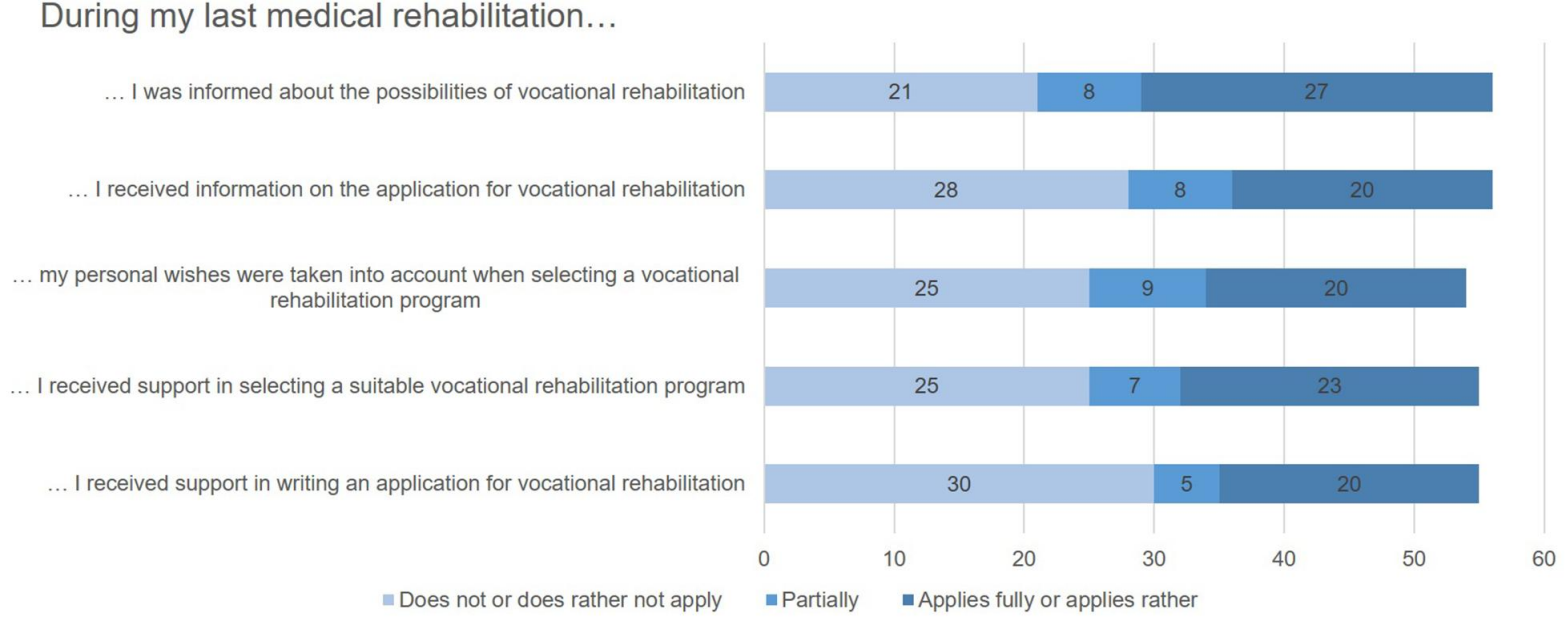
Rehabilitants’ experiences of preparation for vocational rehabilitation during medical rehabilitation (*N* = 54–56).

As illustrated in Figure 1, almost half of the participating rehabilitants reported that they received information about the possibilities of vocational rehabilitation during their stay in a medical rehabilitation clinic. This is also evident from the interview data. For instance, one interviewee reported, “*Then she (the doctor at the medical rehab center) thought back and forth and said: “Well, there's something else where the pension insurance will pay for retraining.” I had to go to the social services, (…) and that's what they suggested to me.”* (4;13). However, more than half of the participants denied receiving support in applying for vocational rehabilitation. For instance, another interviewee stated, “*(I) applied for everything on my own, including the current program. I didn't really have any support there.”* (7;72)

### 3.3 Supportive stakeholders

**Figure 2:**
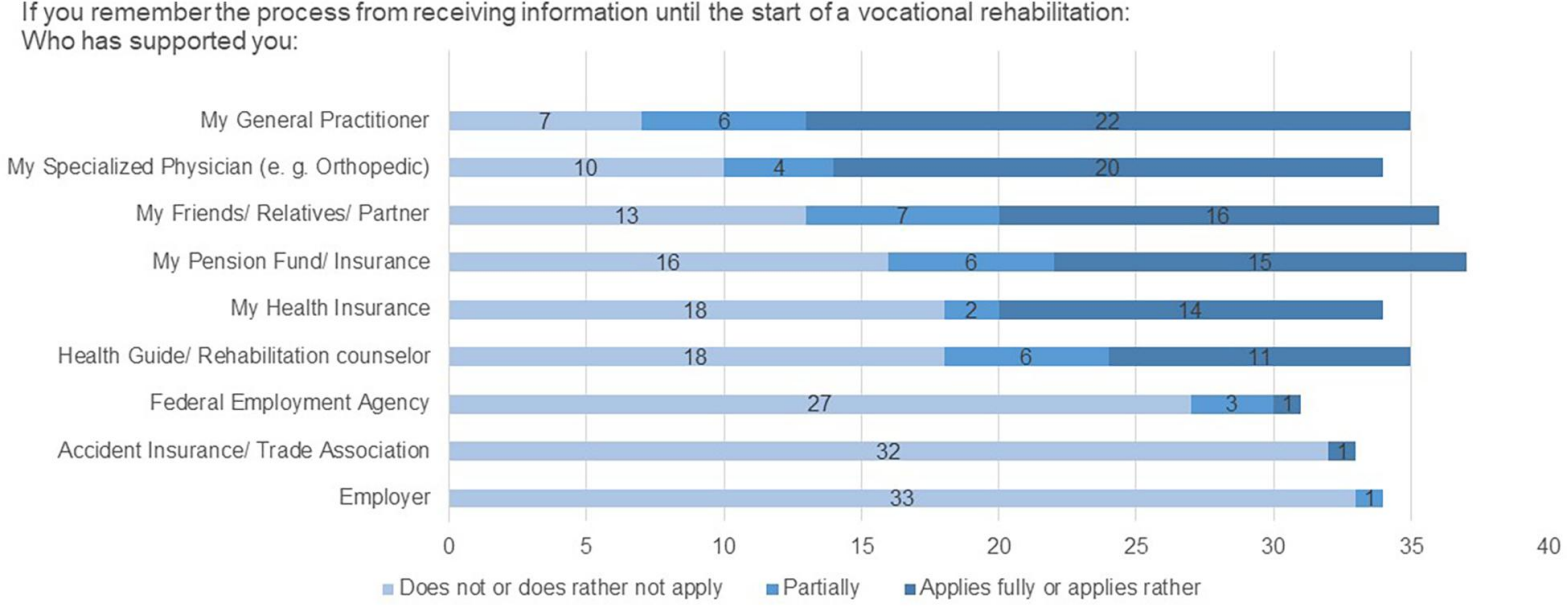
Perceived support by different stakeholders (*N* = 31–37).

When it comes to support by different professional as well as non-professional stakeholders, physicians are reported as supportive in the process of applying for vocational rehabilitation by roughly 60% of the replying participants (see Figure 2).

One interviewee describes the process the following: “*I went on sick leave and my GP had already mentioned this application (for vocational rehabilitation). (…) She had looked after me over the years. She knew my diagnosis and also said: “It's not getting any better, why don't you do it?” Yes, that got the ball rolling.”* (2;60)

97% of the participating survey participants perceive their former or current employers as unsupportive, even though employers are legally required to try to reintegrate employees with chronic health conditions. Concerning lacking support from her employer, a big logistics company, one interviewee recounts: “*I've had these Corporate Integration Management meetings and (…) these tips should actually already be available then. If you have an illness and can no longer do (previous work), that you could have been supported. I have to say, that would be the intersection. I don't know if that's the case at several companies; I can only speak for myself, the topic was not raised at all.”* (2;58)

Additionally, the federal employment agency, which is one of the two major funders of vocational rehabilitation, is not described as not very supportive by 80% of the participants. This lack of support is also noted by 94% for accident insurances/ trade associations.

Several interviewees perceived the lacking cooperation of different professional stakeholders, especially institutions, as detrimental to their overall rehabilitation process, making it very difficult to navigate. One interviewee states “*They don't cooperate, all these institutions. (…) I always had to send to the pension insurance, send it to the health insurance, then back to the employment agency.”* (5;129) Another describes with even more emphasis “*At some point, I got so tired of it. You went from A to B and from B to C and from C back to A. So you're always being pushed back and forth like a ping-pong ball.”* (4;63)

### 3.4 Autonomy

**Figure 3:**
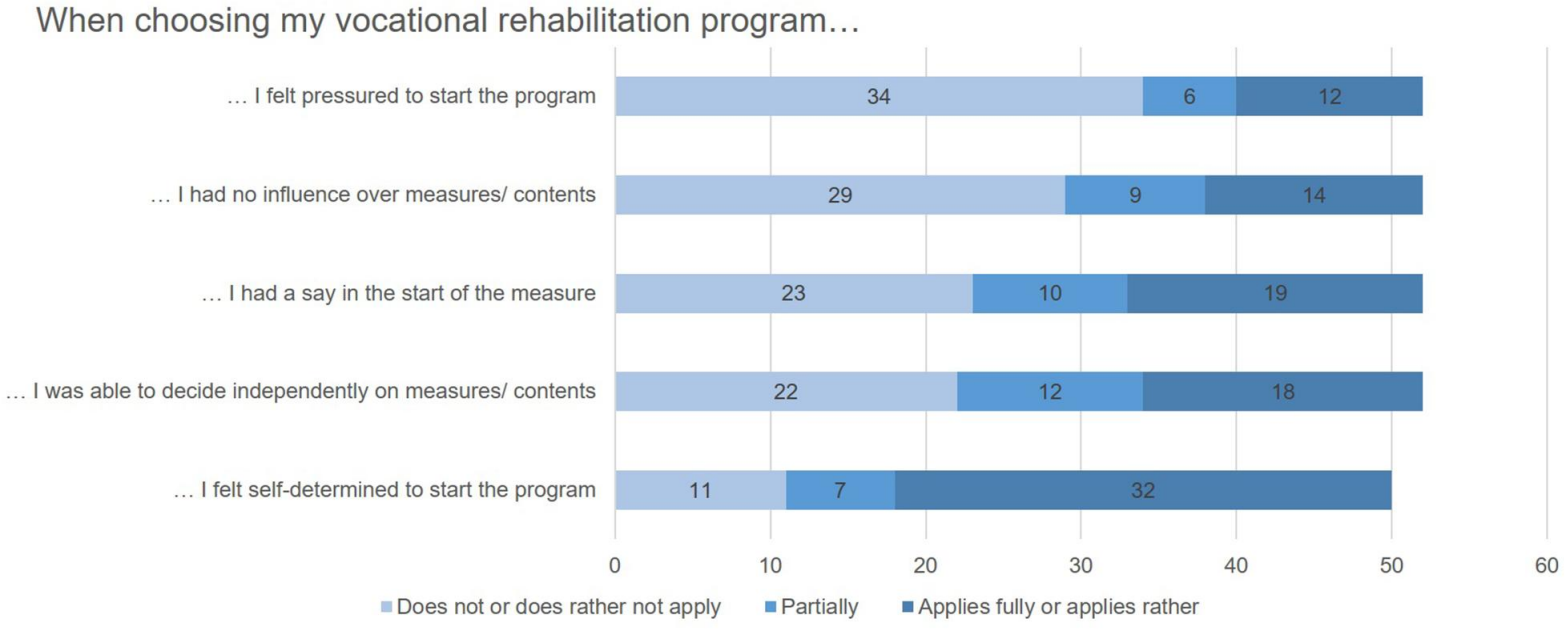
Perceptions of self-determination in the selection of vocational rehabilitation programs (*N* = 50–52).

As can be seen in Figure 3, almost two-thirds of the participants report having felt self-determined to start their vocational rehabilitation program. When it comes to the starting date and deciding on which programs they would start, the rehabilitants describe mixed experiences. Also in the interviews, the rehabilitants often report they had to wait some time for an open spot. Several interviewees would have preferred to start a retraining program right away, but instead had to undergo preparatory programs, which are intended to increase their resilience or refresh basic skills like computer use.

It has to be noted that while the majority did not, around 25% of the participants report having felt like they had no influence over their programs and even pressured to start the program.

The following interview excerpt illustrates how perceptions of self-determination and external control can coexist.

EW: If you think back to the whole process, to what extent did you feel self-determined?

Rehabilitant: Not much. (…) Well, in these programs, (…) no, I felt very externally controlled in many ways. Simply out of the fear of not achieving my goal.

EW: But the fact that you set the goal yourself.. Nobody told you that you had to do it, did they?

Rehabilitant: No, no, no. That's right. (7;89-92)

### 3.5 Navigating the social system on my own

Another theme that emerged from and often recurred in the interviews was “doing it on my own”. Several interviewees recounted not having had much help from professionals, having independently navigated their way through the complex rehabilitation and health care system, and having stood their ground. One interviewee recounts that she had fought for her rehabilitation, knowing from her own professional background that there should be a program tailored to her needs as a patient and single mother.

“So if I hadn't taken the initiative and known that there was still a possibility somehow, then.. So others who don't come from the therapeutic field, I think they would have given up and stayed at home. It was all my doing. (…) Someone else would probably have given up long ago.” (5; 29-31)

These participants also brought this up as a motivation for why they took part in an interview and expressed the hope that by sharing their experiences they might contribute to making it less difficult for future rehabilitants.

Many interviewees expressed that their sense of justice and their expectation from the welfare state—believing it should not abandon individuals—empowered them to navigate the difficulties of the German health and rehabilitation system, despite its challenges. One interviewee, who even took her fight for rehabilitation to court, stated: “*I've always been a justice person; I can't reconcile it with my sense of justice. It can't be right that you get dropped. I worked for years, and if I hadn't taken care of it myself, I would have gone under now.”* (1; 163)

## 4 Discussion

### 4.1 Strengths and limitations

With our study, we aimed to explore rehabilitants' expectations and experiences concerning support and autonomy at the transition from medical to vocational rehabilitation.

The convergent parallel mixed-methods design allowed us to explore both the subjective experiences and simultaneously test their distribution. Our participants show similar sociodemographic characteristics to those of other studies on rehabilitants in vocational rehabilitation (28).

Originally, both medical and vocational rehabilitation participants were intended to participate in the study, allowing for both prospective and retrospective perspectives on the transition from medical to vocational rehabilitation. However, despite extensive efforts and promotion across three different clinics, very few medical rehabilitants completed the survey, and no medical rehabilitants participated in the interviews. Consequently, we decided to focus exclusively on the retrospective views of the transition experienced by vocational rehabilitants. The topic of this study appears to be more interesting for vocational rehabilitants, possibly because they are already more recovered than medical rehabilitants or because they have already successfully mastered the transition. It is plausible that the latter group does not yet consider the topic of future employment or has not been informed about the possibilities of vocational rehabilitation.

For vocational rehabilitants, the study also provided an opportunity for reflection on their transition experiences. However, this retrospective approach may introduce a memory bias, highlighting the need for further research that includes participants who are currently undergoing this transition or who view it from a prospective perspective. Additionally, due to the relatively small number of interviews, despite recurring themes, we do not assume our data are fully saturated.

Additionally, there may be another bias in our findings, as only those individuals who successfully navigated this transition participated in the study. Those whose applications were rejected or who struggled with the transition were excluded from the analysis. This could have potentially excluded very vulnerable groups.

Regarding the emerging theme of navigating this transition independently, it is essential to consider that participants may tend to portray themselves as highly active and self-reliant, as this self-portrayal is likely to enhance their self-esteem.

### 4.2 General discussion

Most rehabilitants report being informed about vocational rehabilitation during medical rehabilitation, but fewer report receiving concrete support, for instance, in applying for a selected program. Concerning supportive stakeholders, many perceived their general or specialized physician as helpful, whereas the employers, the federal employment agency and accident insurances were perceived as not very supportive. Regarding the federal employment agency and accident insurances, it has to be noted that these are formally not responsible for all rehabilitants. However, as important funders of vocational rehabilitation, the lack of support attributed to them is still noteworthy. Several participants mentioned in the interviews that they would have liked more support from their employers, showing that relevant stakeholders are not just within the rehabilitation or health care system. More generally speaking, it can be inferred that ensuring continuity of care at this transition through multi-professional teamwork is not practiced as needed yet.

Regarding autonomy or self-determination, the majority of the participants felt self-determined to start their vocational rehabilitation program. They describe slightly less perceived autonomy in choosing the starting date and the exact program. One-quarter of the participants even state having felt pressured to start a vocational rehabilitation or like they had no say in deciding which program they would start. While the latter is only a minority of the study participants, it would still be a clear violation of the German participation act which aims to improve the self-determination of persons with disabilities (15) as well the German Social code which grants rehabilitants' right to choose the institution in which they want to take their rehabilitation (20). In their scoping review regarding autonomy in medical rehabilitation, Klemmt and colleagues find autonomy-endangering aspects, among other areas, in the rehabilitation system and rehabilitation institutions which fits with our participants reporting that their self-determination was in parts restricted because of systemic and institutional requirements (17). Studies show that vocational rehabilitation and return-to-work interventions which satisfy rehabilitants' needs for self-determination and support them in their autonomy have the potential of leading to higher return-to-work rates, motivation and psychosocial outcomes (29, 30). In general, putting our findings regarding autonomy and self-determination in the context of patient adherence points out that autonomy and shared decision-making are not some state which is at some point fully achieved, but should continuously be seen as goals of care (16).

An emerging theme was the participants’ perception of navigating the social security system on their own without receiving much professional support.

Both our quantitative and qualitative results suggest that continuity of care is partially provided by family or specialized physicians working in private practices. Multidisciplinary teamwork, which can also ensure continuity of care, does not seem to achieve this goal in this setting due to lacking collaboration of different stakeholders and institutions (4).

Our findings further corroborate the already established knowledge that transitions of care can pose a challenge, especially to vulnerable patients and extend this knowledge to the transition between medical and vocational rehabilitation (1–3). What appears to be specific for this transition is the common change between funders and responsible actors and that there is no systematic aftercare or transition management, requiring a lot of initiative by the rehabilitants. Our emergent finding that participants perceive the German rehabilitation system as requiring high navigation competencies from them and as not having much help with this, is in line with many other studies emphasizing the navigation requirements of health and social security systems (31–34).

There are studies that point out potential solutions for the challenges associated with transitions of care. Some of these solutions could also be relevant to the transition between medical and vocational rehabilitation. For example, the Transitional Care Model proposes a nurse-led, team-based case management system that is supported by good evidence (35). In the German rehabilitation context, it is possible that a different profession other than nurses may need to assume responsibility for this. Case managers and/ or rehabilitation counselors both are meant to take this role. However, a study examining the effectiveness of employment-focused case management on helping individuals with substance use disorders return to work found no significant difference in return-to-work rates when compared to standard care (36). Possible solutions already partially in place are single contact points for employers. In general, employers, accident insurances and the federal employment agency as the stakeholders perceived as least important could be held accountable and the long-term benefits of supporting rehabilitants at this transition could be pointed out to them. Another possibility could be digitals tools, such as websites which provide information on different rehabilitation services and on ways to apply and help people navigate different institutions and stakeholders. In general, potential solutions have to be carefully tailored to the specific needs, tested and readjusted. This need is currently already seen by some vocational rehabilitation actors.

### 4.3 Conclusion

Overall, we found that both support and autonomy are vital for rehabilitants at the transition from medical to vocational rehabilitation. While many rehabilitants described feeling adequately supported by their outpatient physicians, they still feel that they are often left alone with having to navigate this transition. Employers do not currently provide sufficient support. Long-term employees, in particular, expressed the expectation of receiving more support and information on the possibilities of reintegration. Also, other stakeholders like the federal employment agency, accident insurances, pension funds, and health insurances as the major actors in the rehabilitation system, could improve their support and their cooperation. The lack of cooperation between different stakeholders and institutions makes it unnecessarily complicated for rehabilitants. With respect to the kind of support that is warranted, especially concrete help in, for instance, filling out applications could be improved.

Regarding autonomy, while only a few people reported feeling pressured or having no influence over the selection of their program, there is still room for improvement to put the ideal of self-determination into practice. A promising approach for this could be openly discussing expectations and preferences with rehabilitants, knowing they might not always be accommodated.

For future research, it would be desirable to include not only the retrospective view on the transition from medical to vocational rehabilitation but also the prospective view. It would be even better to monitor rehabilitants' experiences continuously, for instance in a go-along study.

## Data Availability

The datasets for this article cannot be made publicly available due to German data protection regulations. Requests to access the datasets can be directed to the corresponding author.
