# Supplementary material for: The transition from medical to vocational rehabilitation in Germany: an exploratory mixed methods study on rehabilitants’ experiences
Source: Front Rehabil Sci. 2026 Aug 5;7:1658258. doi: 10.3389/fresc.2026.1658258 (PMC13486271; doi:10.3389/fresc.2026.1658258)
Supplement: Supplementary file 1 [file supplementaryfile1.pdf]

MHB ZVF-BB Zentrum für Versorgungsforschung Brandenburg

FRAGEBOGEN
REHA-Routes

(an Personen in der beruflichen Rehabilitation,
die vorher eine medizinische Reha durchlaufen haben)

Sehr geehrte Teilnehmerin,
sehr geehrter Teilnehmer,

wir freuen uns über Ihr Interesse an unserer Studie! Wir möchten gerne herausfinden, wie Wege von einer medizinischen in eine berufliche Rehabilitation gut vorbereitet und begleitet werden können. Ihre Erfahrung hilft uns, den Prozess besser zu verstehen. Bitte füllen Sie den nachfolgenden Fragebogen aus und senden Sie ihn im beiliegenden Umschlag an uns zurück.
Wir möchten Sie darauf hinweisen, dass Sie sich jederzeit dazu entscheiden können, einzelne Fragen des Fragebogens nicht zu beantworten. Berücksichtigen Sie jedoch, dass für die wissenschaftliche Auswertung die vollständige Beantwortung aller Fragen wichtig ist. Ihre Angaben stehen ausschließlich den an der Studie beteiligten Wissenschaftlern und Wissenschaftlerinnen in anonymer Form zur Verfügung. Das heißt, niemand kann aus Ihren Angaben Rückschlüsse auf Ihre Person ziehen.

Bitte beachten Sie beim Ausfüllen folgende **Hinweise:**

- *Verwenden Sie bitte einen Kugelschreiber*
- *Kreuzen Sie die Kästchen deutlich an:* ☒
- *Bei versehentlicher Falschauswahl füllen Sie das Kästchen bitte ganz aus* ■ *und kreuzen Ihre richtige Auswahl erneut an:* ☒

Vielen Dank für Ihre Unterstützung!

A – Soziodemographie und Krankheitsbiografie					
1	Wie alt sind Sie?	|__|__| (Bitte Alter in Jahren eintragen)			
2	Welches Geschlecht haben Sie?	☐ Weiblich<br>☐ Divers		☐ Männlich<br>☐ Keine Angabe	
3	Bitte geben Sie Ihren derzeitigen Familienstand an.	☐ alleinstehend<br>☐ verheiratet<br>☐ feste Partnerschaft		☐ getrennt lebend<br>☐ geschieden<br>☐ verwitwet	
4	Wie viele Personen leben in Ihrem Haushalt?	|__|__| (Anzahl der Personen, Sie selbst eingeschlossen)			
5	Haben Sie Kinder	☐ Nein ☐ Ja → |__|__| Kinder			
6	Pflegen Sie Angehörige (z.B. Kinder, Menschen mit Behinderung, ältere Personen)?	☐ Nein<br>☐ Ja, bei mir im Haushalt lebende Person(en)<br>☐ Ja, bei mir <u>nicht</u> im Haushalt lebende Person(en)			
7	Wie viele Einwohner hat Ihr derzeitiger Wohnort?	Weniger als 5.000 Einwohner, ohne Nähe zur Stadt	☐	Weniger als 5.000 Einwohner, in der Nähe zur Stadt	☐
		5.000 bis 20.000 Einwohner	☐	20.000 bis 100.000 Einwohner	☐
		Mehr als 100.000 Einwohner	☐		
8	Wie hoch ist Ihr durchschnittliches monatliches Netto-Einkommen?	Unter 1.000 €	☐	1.000 – 1.500 €	☐
		1.500 – 2.500 €	☐	Mehr als 2.500 €	☐
		Keine Angabe	☐		
9	In welchem Bundesland leben Sie?	________________________________			
10	Sind Sie in Deutschland geboren?	Ja	☐	Nein	☐
11	Was ist Ihr höchster Schulabschluss?	Hauptschule	☐	Mittlere Reife	☐
		Polytechnische Oberschule	☐	Abitur	☐
		Kein Schulabschluss	☐	Sonstiges	☐
12	Was ist Ihr höchster beruflicher Bildungsabschluss	Berufsausbildung	☐	Meister / Fachwirt	☐
		Studium	☐	Sonstiges	☐
		Kein beruflicher Abschluss	☐		

13	Was ist Ihr derzeitiger Erwerbsstatus? *(Mehrfachantworten möglich)*	Selbstständig ☐ Angestellt ☐<br>Arbeitssuchend ☐ Arbeitsunfähig ☐<br>Rente ☐ In Aus-/Weiterbildung/ Studium ☐<br>Sonstiges ☐
14	Als Sie zuletzt erwerbstätig waren, wie viele Stunden haben Sie gearbeitet?	☐ Ganztags (mind. 6 Stunden pro Tag bzw. mind. 30 Stunden pro Woche)<br>☐ Halbtags (3 bis 6 Stunden pro Tag bzw. mind. 15 Stunden pro Woche)<br>☐ Weniger als halbtags (bis zu 3 Stunden pro Tag bzw. bis zu 15 Stunden pro Woche)
15	In welcher Branche arbeiten Sie?	☐ Land-, Forst- und Gartenbau<br>☐ Industrie und Fertigung<br>☐ Bau und Ausbau<br>☐ Lebensmittel- und Gastgewerbe<br>☐ Gesundheit<br>☐ soziale und kulturelle Dienstleistungen, Bildung<br>☐ Handel<br>☐ Unternehmensbezogene Dienstleistungen<br>☐ IT- und naturwissenschaftliche Dienstleistungen<br>☐ Sicherheit, Verkehr und Logistik<br>☐ Reinigung<br>☐ Sonstiges, und zwar ______________________________
16	In welchem Bereich liegt Ihr hauptsächliches Gesundheitsproblem? *(Mehrfachantworten möglich)*	Herz-Kreislauf-System ☐ Psyche ☐<br>Muskel-Skelett-System (z.B. chronische Rückenschmerzen) ☐ Sucht ☐<br>Neubildung (Krebs) ☐ Verdauungssystem ☐<br>Nervensystem (z.B. ADHS, Alzheimer) ☐ Haut (z.B. Neurodermitis, Schuppenflechte) ☐<br>Stoffwechsel (z.B. Diabetes) ☐ Atmungssystem (z.B. Asthma, Lungenkrebs) ☐<br>Infektionen ☐ Sonstiges ☐
17	Waren Sie vor Beginn der Maßnahme krankgeschrieben?	☐ Nein<br>☐ Ja, und zwar → |__|__|__| Wochen
18	Wie lange leiden Sie bereits an Ihrem Gesundheitsproblem?	|__|__| (Jahre) |__|__| (Monate)

	**Wie stark fühlen Sie sich momentan durch folgende Sorgen und Probleme belastet?**	Nicht belastet	Etwas belastet	Stark belastet
**19**	Sorgen über Ihre Gesundheit	☐	☐	☐
**20**	Schwierigkeiten mit dem Ehepartner, Lebensgefährten oder Freunden	☐	☐	☐
**21**	Belastungen durch die Versorgung von Kindern, Eltern oder anderen Familienangehörigen	☐	☐	☐
**22**	Finanzielle Probleme/ Sorgen	☐	☐	☐
**23**	Niemanden zu haben, mit dem man Probleme besprechen kann	☐	☐	☐
**24**	Schulden	☐	☐	☐
**25**	Etwas Schlimmes, was vor kurzem passiert ist	☐	☐	☐
**26**	Gedanken an belastende Ereignisse aus der Vergangenheit	☐	☐	☐

B - Arbeitsfähigkeit

**27**	Wegen meines Gesundheitszustands bin ich in meiner Arbeitsfähigkeit beeinträchtigt.							
	☐ Gar nicht beeinträchtigt	☐	☐	☐	☐	☐	☐	☐ Sehr stark beeinträchtigt
**28**	Wegen meines Gesundheitszustands bin ich in der Haushaltsführung (Putzen, Aufräumen, Einkaufen, Kochen, Kinder betreuen, Rechnungen bezahlen) beeinträchtigt.							
	☐ Gar nicht beeinträchtigt	☐	☐	☐	☐	☐	☐	☐ Sehr stark beeinträchtigt
**29**	Wegen meines Gesundheitszustands bin ich in meinen sozialen Freizeitaktivitäten (mit anderen Leuten, z.B. Partys, Bars, Clubs, Ausflüge, Besuche, Verabredungen, Heimunterhaltung) beeinträchtigt.							
	☐ Gar nicht beeinträchtigt	☐	☐	☐	☐	☐	☐	☐ Sehr stark beeinträchtigt
**30**	Wegen meines Gesundheitszustands bin ich in meinen privaten Freizeitaktivitäten (alleine ausgeübt, so wie Lesen, Gartenarbeit, Sammeln, Nähen, alleine Spazierengehen) beeinträchtigt.							
	☐ Gar nicht beeinträchtigt	☐	☐	☐	☐	☐	☐	☐ Sehr stark beeinträchtigt
**31**	Wegen meines Gesundheitszustands bin ich in meiner Fähigkeit, enge Beziehungen mit anderen einzugehen und aufrechtzuerhalten, inklusive der Personen, mit denen ich zusammenwohne, beeinträchtigt.							
	☐ Gar nicht beeinträchtigt	☐	☐	☐	☐	☐	☐	☐ Sehr stark beeinträchtigt

C – Im Folgenden geht es um Ihre letzte medizinische Reha-Maßnahme.

32 Meine letzte medizinische Reha-Maßnahme hat …

- ☐ ambulant stattgefunden.
- ☐ teilstationär stattgefunden.
- ☐ stationär stattgefunden.

33 Meine letzte medizinische Reha-Maßnahme war…

- ☐ eine rein medizinische Rehabilitation.
- ☐ eine arbeitsplatzbezogene Rehabilitation (z.B. ABMR oder MBOR).
- ☐ Ich bin mir nicht sicher.

34 Meine letzte medizinische Reha-Maßnahme war…

- ☐ vor weniger als drei Monaten.
- ☐ vor drei bis sechs Monaten.
- ☐ vor mehr als sechs Monaten.

	**Die Inhalte meiner letzten medizinischen Reha-Maßnahme…**	Trifft überhaupt nicht zu	Trifft eher nicht zu	Teils, teils	Trifft eher zu	Trifft voll und ganz zu
**35**	…passten zu meinen gesundheitlichen Zielen und Bedürfnissen.	☐	☐	☐	☐	☐
**36**	…unterstützten mich darin, meine persönlichen Ziele zu erreichen.	☐	☐	☐	☐	☐
**37**	…berücksichtigten meine gesundheitlichen Einschränkungen.	☐	☐	☐	☐	☐
**38**	…berücksichtigten seelische Aspekte meines Lebens.	☐	☐	☐	☐	☐
**39**	…berücksichtigten soziale Aspekte meines Lebens.	☐	☐	☐	☐	☐
**40**	…waren zu komplex und überforderten mich.	☐	☐	☐	☐	☐
**41**	…waren für mich nicht neu und unterforderten mich.	☐	☐	☐	☐	☐
**42**	…halfen mir dabei, neues gesundheitliches Wissen zu erwerben.	☐	☐	☐	☐	☐
**43**	…halfen mir dabei, neue Fähigkeiten im Umgang mit meiner Erkrankung zu erwerben.	☐	☐	☐	☐	☐
	**Während meiner letzten medizinischen Reha-Maßnahme…**	Trifft überhaupt nicht zu	Trifft eher nicht zu	Teils, teils	Trifft eher zu	Trifft voll und ganz zu
**44**	…wurde ich über die Möglichkeiten einer beruflichen Rehabilitation informiert.	☐	☐	☐	☐	☐
**45**	…erhielt ich Unterstützung darin, eine geeignete berufliche Reha-Maßnahme zu bestimmen.	☐	☐	☐	☐	☐

	**Während meiner <u>letzten medizinischen Reha-Maßnahme</u>…**	Trifft überhaupt nicht zu	Trifft eher nicht zu	Teils, teils	Trifft eher zu	Trifft voll und ganz zu
**46**	…wurden meine persönlichen Wünsche bei der Wahl geeigneter beruflicher Reha-Maßnahmen berücksichtigt.	☐	☐	☐	☐	☐
**47**	…erhielt ich <u>Informationen</u> zur Antragstellung auf berufliche Reha.	☐	☐	☐	☐	☐
**48**	…erhielt ich konkrete <u>Hilfestellungen</u> bei der Antragstellung zur beruflichen Reha.	☐	☐	☐	☐	☐
**49**	…wurde ich gut auf die Zeit nach der medizinischen Reha vorbereitet.	☐	☐	☐	☐	☐
	**Wenn Sie sich rückblickend an Ihre <u>letzte medizinische Reha</u> erinnern: Durch die Teilnahme…**	Trifft überhaupt nicht zu	Trifft eher nicht zu	Teils, teils	Trifft eher zu	Trifft voll und ganz zu
**50**	…fühlte ich mich gut auf zukünftige berufliche Herausforderungen vorbereitet.	☐	☐	☐	☐	☐
**51**	…konnte ich positiv in meine berufliche Zukunft blicken.	☐	☐	☐	☐	☐
**52**	…konnte ich positiv in meine private Zukunft blicken.	☐	☐	☐	☐	☐
**53**	…konnte ich positiv in meine gesundheitliche Zukunft blicken.	☐	☐	☐	☐	☐

D - Zufriedenheit

**54**	Wie zufrieden waren Sie insgesamt mit der <u>Vorbereitung</u> auf die <u>berufliche</u> Reha-Maßnahme?							
	☐ Gar nicht zufrieden	☐	☐	☐	☐	☐	☐	☐ Sehr zufrieden
**55**	Wie zufrieden waren Sie insgesamt mit Ihrer <u>medizinischen</u> Reha-Maßnahme?							
	☐ Gar nicht zufrieden	☐	☐	☐	☐	☐	☐	☐ Sehr Zufrieden
**56**	Inwiefern würden Sie die <u>medizinische</u> Reha-Maßnahme <u>weiterempfehlen</u>?							
	☐ Überhaupt nicht	☐	☐	☐	☐	☐	☐	☐ Auf jeden Fall
**57**	Wie zufrieden sind Sie insgesamt mit Ihrer <u>beruflichen</u> Reha-Maßnahme?							
	☐ Gar nicht zufrieden	☐	☐	☐	☐	☐	☐	☐ Sehr zufrieden
**58**	Inwiefern würden Sie die <u>berufliche</u> Reha-Maßnahme <u>weiterempfehlen</u>?							
	☐ Überhaupt nicht	☐	☐	☐	☐	☐	☐	☐ Auf jeden Fall

F - Berufliche Rehabilitation
Bitte beziehen Sie Stellung zu den folgenden Aussagen. Beziehen Sie sich dabei bitte immer auf die berufliche Reha-Maßnahme, an der Sie aktuell teilnehmen.

**59**	Wie lange hat es gedauert, bis Sie Ihre Maßnahme beginnen konnten? (vom Antrag bis zum Antritt)	ca. |__|__| Monate |__|__| Wochen

	Bitte kreuzen Sie an, inwiefern Sie den folgenden Aussagen zustimmen. **Bei der Wahl meines beruflichen Reha-Programmes…**	Trifft überhaupt nicht zu	Trifft eher nicht zu	Teils, teils	Trifft eher zu	Trifft voll und ganz zu
**60**	…fühlte ich mich selbstbestimmt, die Maßnahme anzutreten.	☐	☐	☐	☐	☐
**61**	…fühlte ich mich unter Druck gesetzt, die Maßnahme anzutreten.	☐	☐	☐	☐	☐
**62**	…konnte ich keinen Einfluss auf vermittelte Maßnahmen nehmen.	☐	☐	☐	☐	☐
**63**	…konnte ich eigenverantwortlich über vermittelte Maßnahmen entscheiden.	☐	☐	☐	☐	☐
**64**	…konnte ich über den Beginn der Maßnahme mitbestimmen.	☐	☐	☐	☐	☐
	**Die Inhalte der beruflichen Reha-Maßnahme…**	Trifft überhaupt nicht zu	Trifft eher nicht zu	Teils, teils	Trifft eher zu	Trifft voll und ganz zu
**65**	…passen zu meinen beruflichen Zielen und Bedürfnissen.	☐	☐	☐	☐	☐
**66**	…unterstützen mich darin, meine persönlichen Ziele zu erreichen.	☐	☐	☐	☐	☐
**67**	…berücksichtigen meine bisherigen beruflichen Kenntnisse.	☐	☐	☐	☐	☐
**70**	…berücksichtigen meine gesundheitlichen Einschränkungen.	☐	☐	☐	☐	☐
**71**	…berücksichtigen seelische Aspekte meines Lebens.	☐	☐	☐	☐	☐
**72**	…berücksichtigen soziale Aspekte meines Lebens.	☐	☐	☐	☐	☐

	**Die Inhalte der beruflichen Reha-Maßnahme…**	Trifft überhaupt nicht zu	Trifft eher nicht zu	Teils, teils	Trifft eher zu	Trifft voll und ganz zu
**73**	…haben einen angemessenen Umfang.	☐	☐	☐	☐	☐
**74**	…sind zu komplex und überfordern mich.	☐	☐	☐	☐	☐
**75**	…sind für mich nicht neu und unterfordern mich.	☐	☐	☐	☐	☐

	**Die Inhalte der beruflichen Reha-Maßnahme…**	Trifft überhaupt nicht zu	Trifft eher nicht zu	Teils, teils	Trifft eher zu	Trifft voll und ganz zu
**76**	…helfen mir dabei, neues berufliches Wissen zu erwerben.	☐	☐	☐	☐	☐
**77**	… helfen mir dabei, meine beruflichen Fähigkeiten weiterzuentwickeln.	☐	☐	☐	☐	☐
**78**	… helfen mir dabei, neues Selbstbewusstsein über meine eigenen Fähigkeiten zu erlangen.	☐	☐	☐	☐	☐
	**Die beruflicheReha-Maßnahme…**	Trifft überhaupt nicht zu	Trifft eher nicht zu	Teils, teils	Trifft eher zu	Trifft voll und ganz zu
**79**	…ist für mich örtlich gut erreichbar.	☐	☐	☐	☐	☐
**80**	…lässt sich gut in meinen privaten Alltag integrieren.	☐	☐	☐	☐	☐
**81**	…erlaubt mir, meinen täglichen Verpflichtungen weiterhin nachzukommen.	☐	☐	☐	☐	☐
**82**	…erlaubt mir, mich weiterhin um meine Angehörigen zu kümmern. *(sofern zutreffend, sonst bitte frei lassen)*	☐	☐	☐	☐	☐
**83**	…lässt sich gut in mein Berufsleben integrieren. *(sofern zutreffend, sonst bitte frei lassen)*	☐	☐	☐	☐	☐
	**Durch die Teilnahme an der beruflichen Reha-Maßnahme…**	Trifft überhaupt nicht zu	Trifft eher nicht zu	Teils, teils	Trifft eher zu	Trifft voll und ganz zu
**84**	…fühle ich mich gut auf künftige berufliche Herausforderungen vorbereitet.	☐	☐	☐	☐	☐
**85**	…kann ich positiv in meine berufliche Zukunft blicken.	☐	☐	☐	☐	☐
**86**	…kann ich positiv in meine private Zukunft blicken.	☐	☐	☐	☐	☐

**G - Maßnahmen und Leistungen der beruflichen Rehabilitation**			
**87**	**Welche der folgenden Maßnahmen im Rahmen einer beruflichen Reha nehmen Sie aktuell in Anspruch?**<br><br>**Welche haben Sie ggf. in der Vergangenheit in Anspruch genommen?**	Nehme ich aktuell in Anspruch (Mehrfachauswahl möglich)	Habe ich in der Vergangenheit (Mehrfachauswahl möglich)
	Hilfen zum Erhalt eines Arbeitsplatzes bzw. bei der Jobsuche	☐	☐
	Berufliche Anpassung, Ausbildung, Fortbildung bzw. Weiterbildung	☐	☐
	Berufsvorbereitung (einschließlich Erstausbildung)	☐	☐
	Gründungszuschuss bei Aufnahme einer selbständigen Tätigkeit	☐	☐
	Unterstützung für den Arbeitgeber, um Ihre Beschäftigung zu ermöglichen	☐	☐
	Leistungen in einer anerkannten Werkstatt für behinderte Menschen (WfbM)	☐	☐
	Hilfsmittel und technische Arbeitshilfen	☐	☐
	Andere, und zwar_________________________________	☐	☐
	Ich weiß nicht, wo meine Maßnahme einzuordnen ist.	☐	☐

**H - Unterstützung**						
Rückblick: Wenn Sie sich den Prozess von Informationsbereitstellung bis zum Antritt einer beruflichen Reha erinnern: Wer hat Sie unterstützt?		Trifft überhaupt nicht zu	Trifft eher nicht zu	Teils, teils	Trifft eher zu	Trifft voll und ganz zu
**88**	Mein Hausarzt	☐	☐	☐	☐	☐
**89**	Mein Facharzt (z.B. Orthopädin)	☐	☐	☐	☐	☐
**90**	Mein/e Freunde / Verwandten / Partner	☐	☐	☐	☐	☐
**91**	Die Agentur für Arbeit / das Jobcenter	☐	☐	☐	☐	☐
**92**	Meine Krankenkasse	☐	☐	☐	☐	☐
**93**	Meine Rentenversicherung	☐	☐	☐	☐	☐
**94**	Meine Unfallkasse / Berufsgenossenschaft	☐	☐	☐	☐	☐
**95**	Gesundheitslotse / Reha-Fachberater	☐	☐	☐	☐	☐
**96**	Mein Arbeitgeber	☐	☐	☐	☐	☐
**97**	Sonstige, und zwar: ____					

	**Arbeitgeber:** Bitte beantworten Sie die Fragen nur, wenn Sie sich in einem bestehenden Arbeitsverhältnis befinden.	Trifft überhaupt nicht zu	Trifft eher nicht zu	Teils, teils	Trifft eher zu	Trifft voll und ganz zu
**98**	Mein Arbeitgeber unterstützt mich darin, meinen Arbeitsplatz an meine gesundheitliche Problemlage anzupassen.	☐	☐	☐	☐	☐
**99**	Mein Arbeitgeber unterstützt mich darin, meine Arbeitsaufgaben an meine gesundheitliche Problemlage anzupassen.	☐	☐	☐	☐	☐
**100**	Ich habe das Gefühl, mein Arbeitgeber ist interessiert daran, mich auch weiterhin im Betrieb zu beschäftigen.	☐	☐	☐	☐	☐
**100**	Meine Kollegen unterstützten mich darin, eine meinen Bedürfnissen entsprechende berufliche Reha-Maßnahme anzutreten.	☐	☐	☐	☐	☐

E - Gesundheitsinformation finden, verstehen und beurteilen

Die folgenden Fragen beziehen sich allgemein auf die medizinische Versorgung, nicht nur auf Ihre Reha.

	**Auf einer Skala von sehr einfach bis sehr schwierig: Wie einfach ist es Ihrer Meinung nach im Allgemeinen …**	Sehr schwierig	Ziemlich schwierig	Ziemlich einfach	Sehr einfach
**101**	Informationen über Krankheitssymptome, die Sie betreffen, zu finden?	☐	☐	☐	☐
**102**	Informationen über Therapien für Krankheiten, die Sie betreffen, zu finden?	☐	☐	☐	☐
**103**	herauszufinden, was im Fall eines medizinischen Notfalls zu tun ist?	☐	☐	☐	☐
**104**	herauszufinden, wo Sie professionelle Hilfe erhalten, wenn Sie krank sind? (Hinweis: z.B. Arzt, Apotheker, Psychologe)	☐	☐	☐	☐
**105**	zu verstehen, was Ihr Arzt Ihnen sagt?	☐	☐	☐	☐
**106**	die Packungsbeilagen / Beipackzettel Ihrer Medikamente zu verstehen?	☐	☐	☐	☐
**107**	zu verstehen, was in einem medizinischen Notfall zu tun ist?	☐	☐	☐	☐
**108**	die Anweisungen Ihres Arztes oder Apothekers zur Einnahme der verschriebenen Medikamente zu verstehen?	☐	☐	☐	☐
**109**	zu beurteilen, inwieweit Informationen Ihres Arztes auf Sie zutreffen?	☐	☐	☐	☐
**110**	Vor- und Nachteile von verschiedenen Behandlungsmöglichkeiten zu beurteilen?	☐	☐	☐	☐
**111**	zu beurteilen, wann Sie eine zweite Meinung von einem anderen Arzt einholen sollten?	☐	☐	☐	☐

	**Auf einer Skala von sehr einfach bis sehr schwierig: Wie einfach ist es Ihrer Meinung nach im Allgemeinen…**	Sehr schwierig	Ziemlich schwierig	Ziemlich einfach	Sehr einfach
**112**	zu beurteilen, ob Informationen über eine Krankheit in den Medien vertrauenswürdig sind? (Fernsehen, Internet oder andere Medien)	☐	☐	☐	☐
**113**	mit Hilfe der Informationen, die Ihnen der Arzt gibt, Entscheidungen bezüglich Ihrer Krankheit zu treffen?	☐	☐	☐	☐
**114**	den Anweisungen für die Einnahme von Medikamenten zu folgen?	☐	☐	☐	☐
**115**	im Notfall einen Krankenwagen zu rufen?	☐	☐	☐	☐
**116**	den Anweisungen Ihres Arztes oder Apothekers zu folgen?	☐	☐	☐	☐

Möchten Sie uns noch etwas mitteilen?

____

Vielen Dank für Ihre Unterstützung!
