# Supplementary material for: The transition from medical to vocational rehabilitation in Germany: an exploratory mixed methods study on rehabilitants’ experiences
Source: Front Rehabil Sci. 2026 Aug 5;7:1658258. doi: 10.3389/fresc.2026.1658258 (PMC13486271; doi:10.3389/fresc.2026.1658258)
Supplement: Supplementary file 2 [file supplementaryfile2.pdf]

Leitfrage	Vertiefungsfragen	Checkliste<br>Wurde das erwähnt?
Bitte schildern Sie mir Ihre aktuelle gesundheitliche, berufliche und soziale Situation.	Wie lange liegt Ihre letzte medizinische Reha zurück?<br>Was haben Sie sich von der med. Reha erhofft? Wurden Ihre Erwartungen erfüllt?<br>Hat sich Ihre gesundheitliche Situation durch die med. Reha verbessert?<br>Wie würden Sie Ihre finanzielle und soziale Situation beschreiben?	Medizinische Reha abgeschlossen<br>Aktuelle gesundheitliche Einschränkungen und Prognose<br>Aktuelle berufliche Situation und Prognose<br>Aktuelle soziale Situation und Prognose<br>Emotionale und kognitive Bewertung der Situation
Soweit ich weiß, sind Sie gerade in einer beruflichen Reha-Maßnahme. Bitte erzählen Sie mir darüber.	Wie ist die Maßnahme ausgestaltet?<br>Was sind die Inhalte?<br>Wo/ bei welcher Einrichtung nehmen Sie die in Anspruch?<br>Wer finanziert die Maßnahme?<br>Haben Sie in der Vergangenheit bereits eine ähnliche Maßnahme in Anspruch genommen?	Art, Dauer der Maßnahme<br>Institution, Kostenträger

**Übergang medizinische – berufliche Reha**		
Wie ging es für Sie nach der medizinischen Reha weiter?<br><br>Wie genau war das Vorgehen nach Abschluss der medizinischen Reha bis zum Beginn der beruflichen?	Warum wurde eine berufliche Reha initiiert?<br>Inwiefern waren Sie selbst daran beteiligt?<br>Wer war noch involviert?<br>Wer hat konkret welche Aufgaben übernommen?<br>Inwiefern ist geplant, dass Sie an Ihren derzeitigen Arbeitsplatz zurückkehren bzw. Ihren gelernten Beruf weiter ausüben?	Motive/Gründe zur Aufnahme einer bR<br>Krankenkassen, DRV<br>Arbeitgeber<br>Ärzte<br>Med. Reha-Einrichtung<br>Freunde/Familie

Wo bzw. durch wen sind Sie über die Möglichkeit einer beruflichen Rehabilitation informiert worden?	Wer hat Sie informiert?<br>Inwiefern wurden Sie über den Prozess der Beantragung einer beruflichen Reha informiert?<br>Wurde die Möglichkeit einer beruflichen Reha bereits in der Reha-Klinik mit Ihnen besprochen?<br>Inwieweit fanden Sie die Information zur bR angemessen?	In der medizinischen Reha angesprochen?<br>Formen und Zeitpunkt der Ansprache
Wie wurde entschieden, was für Sie die passende berufliche Reha-Maßnahme ist?	Wie wurde die Entscheidung getroffen?<br>Welche Aspekte haben Ihrer Meinung nach für oder gegen eine solche Maßnahme für Sie gesprochen?<br>Welche anderen Maßnahmen als Ihre derzeitige wurden noch in Betracht gezogen?	WIE der Entscheidungsfindung<br>Motive der Entscheidungsfindung
Wie haben Sie das Vorgehen empfunden?	Inwiefern haben Sie sich selbstbestimmt gefühlt bei der Entscheidung?<br>Warum genau haben Sie sich eher unwohl gefühlt? Was hätte Ihnen geholfen, sich wohler zu fühlen?<br>Was genau trägt dazu bei, dass Sie sich insgesamt wohl fühlen mit dem Vorhaben?<br>Inwiefern haben Sie sich unterstützt gefühlt?	Faktoren für Zufriedenheit<br>Selbstbestimmtheit<br>Unterstützung

**Wenn angestellt:** Inwiefern ist Ihr Arbeitgeber in den Prozess eingebunden?	Wer ist Ihr Ansprechpartner?<br><br>Welche Unterstützungsangebote stellt der Arbeitgeber Ihnen zur Verfügung? Gibt es bei Mitbestimmungsstrukturen/ Interessenvertretung? (ggf. Größe des Arbeitgebers)<br><br>Aus welchen Gründen ist der Arbeitgeber (nicht) eingebunden?	Vorgesetzte<br>Personalabteilung<br>Wiedereingliederung (BEM)<br>Rückkehrgespräche<br>Betriebsmedizin<br>Vorbehalte gegenüber Einbindung des Arbeitgebers<br>Eigeninitiative vs. Arbeitgeberinitiative

**Zufriedenheit mit der beruflichen Rehabilitation**		
Welche Erwartungen hatten Sie an Ihre berufliche Reha?	Was sollte aus Ihrer Sicht vermittelt werden?<br>Was wäre für Sie ein ideales Ergebnis der beruflichen Rehabilitation?<br>Inwiefern werden Ihre Erwartungen bisher erfüllt?<br>Falls Sie bereits früher eine berufliche Reha in Anspruch genommen haben: Inwieweit unterscheiden sich die Maßnahmen?	Hilfestellung – wobei?<br>Umschulungen, Arbeitsplatzumgestaltungen<br>Technische Hilfsmittel
Wie gut lässt sich die berufliche Reha mit Ihrem Alltag vereinbaren?	Wie sieht es mit dem beruflichen Alltag aus?<br>Wie sieht es mit dem privaten Alltag aus?<br>Inwiefern wird auf Ihre gesundheitlichen Einschränkungen Rücksicht genommen?	Erreichbarkeit<br>Online-Maßnahme<br>Dauer & Umfang
**Abschluss:** Wir sind jetzt schon fast am Ende des Interviews. Wenn Sie noch mal an diesen Übergang zwischen medizinischer Reha und beruflicher Reha denken: Was ist Ihnen noch wichtig zu dem Thema?		
